# Genome Sequence of *Arthrobacter* sp. Phage Scuttle

**DOI:** 10.1128/MRA.00577-20

**Published:** 2020-06-25

**Authors:** Melinda Harrison, Matthew D. Mastropaolo, Andrew Conboy, Cabrini University SEA-PHAGES Annotators

**Affiliations:** a Department of Science, Cabrini University, Radnor, Pennsylvania, USA; b Department of Mathematics and Sciences, Neumann University, Aston, Pennsylvania, USA; c Biological Sciences, Lehigh University, Bethlehem, Pennsylvania, USA; Queens College

## Abstract

*Arthrobacter* phage Scuttle was isolated by enrichment from a dry soil sample (collected in Upper Darby, Pennsylvania) on host *Arthrobacter* sp. ATCC 21022. The genome of this phage is 43,729 bp long, has a GC content of 61.1%, and has 61 annotated protein-coding genes.

## ANNOUNCEMENT

Bacteriophages are regarded as the “dark matter” of the biosphere, due to their abundance in our environment (1). Bacteriophages play a key role in microbial ecology and have also been suggested as a catalyst in maintaining the genetic variability of the bacterial community (2). Their diversity continues to fascinate researchers in terms of the wide range of bacterial hosts they can infect. *Arthrobacter* spp. are highly resistant soil bacteria that can withstand a multitude of environmental stressors, including starvation, heavy metal toxicity, free radicals, and harmful radiation (3–5). Understanding phages that infect *Arthrobacter* spp. would provide insights into these environmentally resilient bacteria.

We have isolated and characterized a *Siphoviridae* bacteriophage, Scuttle, which infects *Arthrobacter* sp. ATCC 21022 (6). Phage Scuttle was isolated from a dry soil sample by students in the Science Education Alliance-Phage Hunters Advancing Genomics and Evolutionary Science (SEA-PHAGES) program (7) using an enrichment procedure (8). The soil sample was suspended in phage buffer, and the bacteriophage was extracted from the mixture through a 0.22-μm filter. For virus replication, a filtered medium was incubated with *Arthrobacter* sp. ATCC 21022 at 30°C for 24 h. Genomic DNA was isolated using a phenol-chloroform protocol (9).

Sequencing, assembly, and finishing of the genome were performed according to Russell (10). The phage sequencing library was prepared using the New England BioLabs (NEB) Ultra II kit v3 and was sequenced using the Illumina MiSeq platform, generating 150-bp unpaired reads. Raw reads were assembled using Newbler 2.9 (11) with default settings, generating a single contig with a coverage of approximately 5,080-fold. Phage ends were determined as previously described (10) using Consed v29 (12) to check for completeness and accuracy of termini. The genome was annotated using DNAMaster v5.23.3 (http://cobamide2.bio.pitt.edu/computer.htm), with coding sequences predicted by GeneMark v2.5p (13) and Glimmer v3.02b (14); using BLAST (15), HHpred (16), and manual inspection (17), 61 protein-coding genes were identified. Phamerator (18) was used for comparative genomic analysis. All software was used with default settings. No tRNA or transfer-messenger RNA (tmRNA) genes were detected by ARAGORN v1.2.38 (19) or tRNAscan-SE v2.0 (20).

Phage Scuttle contains 61 protein-coding genes mostly transcribed rightward, with 5 protein-coding genes located at the end of the genome which are transcribed leftward. The GC content of phage Scuttle is similar to that of its host *Arthrobacter* sp. ATCC 21022, at 61.1% versus 63.41%, respectively (18, 21). Scuttle contains the typical structural and assembly genes, including the capsid, endolysin, tape measure, terminase, portal, major tail, and minor tail proteins. A RecA-like exonuclease, GP44, and a divergent gene, GP17, that is found in other AK phages were also identified. There are also differences in the gene content between Scuttle and its closest relatives, Dino and Zorro. An extra gene, GP 36, is present in phages Dino and Zorro but is absent from phage Scuttle. Scuttle GP57 (coordinates 41927 to 42262) is absent from phages Dino and Zorro.

### Data availability.

Scuttle is available at GenBank with accession no. MK814749 and SRA accession no. SRX8359893.
